# Exploring the Th2 Response in Obesity and Metabolic Dysfunction-Associated Steatotic Liver Disease (MASLD): A Potential Modulator of the Renin-Angiotensin System (RAS) Pathway in Hypertension Development

**DOI:** 10.3390/life14091080

**Published:** 2024-08-29

**Authors:** Lucía Angélica Méndez-García, Galileo Escobedo, Itzel Baltazar-Pérez, Nydia Angélica Ocampo-Aguilera, José Alfonso Arreola-Miranda, Miguel Angel Cid-Soto, Ana Alfaro-Cruz, Antonio González-Chávez, Aquiles Ranferi Ocaña-Guzmán, Helena Solleiro-Villavicencio

**Affiliations:** 1 Immunometabolism Laboratory, General Hospital of Mexico “Eduardo Liceaga”, Mexico City 06720, Mexico; gescobedog@msn.com (G.E.); itzel.bp.050997@gmail.com (I.B.-P.); angelica.ocampo@alumnos.uacm.edu.mx (N.A.O.-A.); alfonso.arreola@estudiante.uacm.edu.mx (J.A.A.-M.); 2 Genomics Sciences Program, Oncogenomics and Cancer Proteomics Laboratory, Autonomous University of Mexico City, Avenue San Lorenzo 290, Mexico City 03100, Mexico; 3 Sequencing Laboratory, Division of Research Development, National Medical Center “Siglo XXI”, Mexican Social Security Institute, Mexico City 06720, Mexico; cidsoto7@gmail.com; 4 Pathological Anatomy Department, General Hospital of Mexico “Dr. Eduardo Liceaga”, Mexico City 06726, Mexico; analfaro@yahoo.com; 5 Comprehensive Care Clinic for Patients with Diabetes and Obesity (CAIDO), General Hospital of Mexico “Dr. Eduardo Liceaga”, Mexico City 06726, Mexico; antoniogonzalezchavez51@gmail.com; 6 Integrative Immunology Laboratory, National Institute of Respiratory Diseases, Mexico City 14080, Mexico; arocana@iner.gob.mx

**Keywords:** non-alcoholic fatty liver disease, metabolic dysfunction-associated steatotic liver disease, systemic arterial hypertension, cytokines, Th2-response, renin-angiotensin system, obesity

## Abstract

Non-alcoholic fatty liver disease (NAFLD), now referred to as metabolic dysfunction-associated steatotic liver disease (MASLD), is alarmingly increasing alongside the cases of obesity worldwide. MASLD is an underestimated metabolic abnormality closely linked with a higher risk of developing systemic arterial hypertension (SAH). However, the underlying mechanism of association between MASLD and SAH remains unknown. Inflammation may link these two entities by regulating the renin-angiotensin system (RAS). For this reason, in this study, we evaluated the hepatic expression of a cytokine profile and critical molecules in the RAS pathway in patients with morbid obesity and MASLD, both with SAH. We found a statistically significant correlation between ACE levels and the cytokines IL-4, IL-10, and IL-13 of Th2 response. Furthermore, according to a multiple linear regression analysis, the cytokines IL-4 and IL-13 were the best predictors of ACE levels. Moreover, we observed increased hepatic IL-13 expression in patients with morbid obesity, MASLD, and SAH compared to those without SAH. These results allow us to propose, for the first time, that the Th2 response, through regulating the RAS, could play a critical role in developing SAH in individuals with MASLD and obesity.

## 1. Introduction

Nonalcoholic liver disease (NAFLD), currently called metabolic dysfunction-associated steatotic liver disease (MASLD) and systemic arterial hypertension (SAH), are common cardiovascular risk factors that are closely related to obesity and metabolic syndrome (MS) [1]. SAH is a multifactorial disease resulting from the interaction between environmental and genetic risk factors, characterized by a sustained increase in systolic, diastolic, or both blood pressures. In recent decades, SAH has become a public health problem with a prevalence of approximately one-third of the world population [2]. MASLD comprises a spectrum of pathologies ranging from simple steatosis to steatohepatitis (MASH) that can progress to advanced fibrosis, cirrhosis, and finally, hepatocellular carcinoma. Globally, MASLD has emerged as the most prevalent chronic liver disease, affecting approximately 25% of the population. Clinical studies have revealed a reciprocal relationship between these two conditions; SAH is identified as an independent predictor of MASLD, and, in turn, MASLD is associated with a higher risk of developing SAH [3]. However, the underlying association mechanism between MASLD and SAH is still not fully understood.

Evidence indicates that inflammation may be critical in the relationship between MASLD and SAH. During the progression of MASLD, liver-resident macrophages, known as Kupffer cells, release proinflammatory cytokines that circulate throughout the body and cause chronic low-grade systemic inflammation. This inflammation activates the sympathetic nervous system (SNS) and the renin-angiotensin system (RAS), which regulate blood pressure. Therefore, RAS dysregulation could contribute to the development of SAH [1].

The RAS comprises two pathways with opposing effects, contributing to maintaining homeostasis [4]. The classical axis involves the angiotensin-converting enzyme (ACE), angiotensin 2 (Ang 2), and the angiotensin type 1 receptor (AT1R), which are responsible for mediating the biological actions of Ang 2, including vasoconstriction, cell proliferation, pro-fibrotic effects, and inflammation. The alternative axis incorporates angiotensin-converting enzyme 2 (ACE2), predominantly acting on Ang II and converting it into angiotensin 1-7 (Ang 1-7). The latter peptide activates MAS receptors (MasR), eliciting vasodilatory, anti-proliferative, anti-fibrotic, and anti-inflammatory effects [4].

In the pathogenesis and progression of SAH, the influence of RAS is not limited solely to the systemic level; local RAS also plays a crucial role. Concerning hepatic RAS, mounting evidence indicates that heightened activation of the classical RAS axis or suppression of the alternative axis is linked to increased expression of genes specific to lipid oxidation, oxidative stress, and inflammation [5,6,7]. Moreover, the intrarenal RAS plays a central role in the long-term regulation of blood pressure, sodium balance, and extracellular fluid volume. All components of the RAS pathway are found in the kidney, and the accumulation of Ang II in specific renal compartments suggests tissue-specific regulation of this pathway [8].

On the other hand, evidence suggests that RAS could be influenced, at least in part, by inflammatory factors. Studies conducted in animal models of lupus nephritis have shown that antihypertensive drugs, such as captopril, that inhibit ACE can cause a parallel reduction in the protein levels of IL-4 and IL-10 in the spleen. These two cytokines are essential to the Th2 immune response, suggesting a possible regulatory interaction between this response and the RAS system [9]. However, the relationship of the Th2 response with diseases such as MASLD and SAH has yet to be elucidated. We hypothesized that there would be significant differences in the levels of hepatic RAS proteins between subjects with obesity and MASLD with SAH compared to those without SAH. Additionally, the hepatic Th2 response could modulate liver RAS molecules, which may be linked to the development of SAH in patients with obesity and MASLD. Hence, in this study, we assessed the key molecules associated with the classical and alternative pathways of the RAS within the livers of patients diagnosed with morbid obesity and MASLD, along with an analysis of cytokines. We hypothesized that there would be significant differences in the levels of hepatic RAS proteins between subjects with obesity and MASLD with SAH compared to those without SAH. Additionally, the hepatic Th2 response could modulate liver RAS molecules, which may be linked to the development of SAH in patients with obesity and MASLD. Hence, in this study, we assessed the key molecules associated with the classical and alternative pathways of the RAS within the livers of patients diagnosed with morbid obesity and MASLD (with and without SAH), along with an analysis of cytokines.

## 2. Materials and Methods

### 2.1. Patients

In our investigation, we undertook a detailed cross-sectional analysis that involved the inclusion of 71 morbidly obese patients (body mass index ≥ 30 kg/m^2^) of both genders. These patients met the criteria and were slated for elective Roux-en-Y gastric bypass at the Clinic for Patients with Obesity and Diabetes and the General Surgery Department of the General Hospital of Mexico. Each enrolled patient provided signed informed consent, previously approved by the General Hospital of Mexico’s Ethics Committee under project number DI/16/UME/05/048. They consented to donate a 3 g liver sample during the surgical procedure. Samples were immediately embedded in 25 mM Tris-HCl buffer after the biopsy and stored at −80 °C. The samples were collected within four months and then thawed at room temperature before performing assays. It is essential to underscore that our study was conducted meticulously in adherence to the principles articulated in the Declaration of Helsinki of 1964 and its subsequent amendment in 2013.

We diligently collected a wide range of demographic, clinical, and biochemical data from all the enrolled patients. This extensive data included their full names, clinical record numbers, gender, age, body mass index (BMI), the prevalence of type 2 diabetes (T2D) and systemic arterial hypertension (SAH), and serum fasting blood glucose levels, triglycerides (TGS) levels, total cholesterol (TC) levels, low-density lipoproteins (LDL), high-density lipoproteins (HDL), albumin (ALB) levels, alanine aminotransferase (ALT) levels, aspartate aminotransferase (AST) levels, gamma-glutamyl transferase (GGT) levels, alkaline phosphatase (AP) levels, total bilirubin (TB) levels, direct bilirubin (DB) levels, and indirect bilirubin (IB) levels, urea levels, creatinine levels, uric acid levels, prothrombin time (PT), international normalized ratio (INR), activated partial thromboplastin time (aPTT), hemoglobin (Hb) levels, leukocytes (Leuc) and platelets (Pla) counts.

### 2.2. Liver Histopathological Evaluation and MASLD Diagnosis

The liver samples were fixed and embedded in paraffin for histological processing. Subsequently, four μm-thick slices were obtained using a microtome (Leica Biosystems, Deer Park, IL, USA). These slices were stained with hematoxylin-eosin to visualize hepatic cells’ cellular nuclei and cytoplasmic components. The steatosis, lobular inflammation, hepatocyte ballooning, and fibrosis stage were assessed using the Brunt scoring system [10] and staining with Picro-Sirius red and Masson’s trichrome. The diagnosis of MASLD was determined based on validated criteria [11], with individuals meeting the MASLD diagnosis if they had steatosis and at least one of the specified cardiometabolic criteria:

- BMI ≥ 25 kg/m^2^ or waist circumference > 94 cm (M) and 80 cm (F), or an ethnicity-adjusted equivalent.
- Fasting serum glucose ≥ 5.6 mmol/L [100 mg/dL] or 2-h post-load glucose levels ≥ 7.8 mmol/L [140 mg/dL], or HbA1c ≥ 5.7% [39 mmol/mol] or a diagnosis of type 2 diabetes or its treatment.
- Blood pressure ≥ 130/85 mmHg or specific antihypertensive drug treatment.
- Plasma triglycerides ≥ 1.70 mmol/L [150 mg/dL] or lipid-lowering treatment.
- Plasma HDL-cholesterol ≤ 1.0 mmol/L [40 mg/dL] (M) and ≤1.3 mmol/L [50 mg/dL] (F) or lipid-lowering treatment.

### 2.3. Quantification of ACE, ACE2, Ang-1,7, and Ang 2 Hepatic Proteins

Stored liver samples were thawed at room temperature and homogenized in an appropriate lysis buffer containing 500 mM Tris-HCl and a protease inhibitor cocktail (Merck, Darmstadt, Germany). The samples were then centrifuged at 20,800× *g* at 8 °C for 15 min to obtain the supernatant containing the proteins of interest. Protein concentration in the supernatant was measured using the Bradford protein assay (Bio-Rad Laboratories, Inc., Hercules, CA, USA) at 595 nm. To quantify ACE, ACE2, Ang-1,7, and Ang 2 levels, we used commercial sandwich enzyme-linked immunosorbent assay (ELISA) kits (R&D Systems, Minneapolis, MN, USA) according to the manufacturer’s instructions. For the ELISA setup, diluted samples and standards were added to the wells of an ELISA plate pre-coated with specific antibodies for ACE, ACE2, Ang-1,7, and Ang2. Absorbance was measured using a spectrophotometer Microplate Reader PKL PPC 142 (Paramedical PKL, Salerno, Italy), and data analysis included the preparation of a standard curve and interpolating protein concentrations from the curve.

### 2.4. Quantification of Hepatic Cytokines

The quantification of cytokines TNF-α, IL-6, IL-4, IL-13, and IL-10 from liver sample supernatants was performed using a LUMINEX assay with the Milliplex Human Cytokine/Chemokine Magnetic Bead Panel (Millipore, Burlington, MA, USA) following the manufacturer’s instructions. The plate was read using the Magpix 200 instrument (Millipore, Burlington, MA, USA) with MILLIPLEX^®^ Analysis 5.1 software (Millipore, Burlington, MA, USA). This method allowed for the precise quantification of cytokine levels in liver samples, facilitating the assessment of inflammatory responses.

### 2.5. Statistics

Upon conducting the Shapiro-Wilk test to assess the normality of the data, the Kruskal-Wallis test was employed to compare more than two groups. The Mann-Whitney U test was utilized to compare the two groups. Subsequently, Spearman’s correlation coefficients were computed to analyze the relationships between biochemical, clinical, and anthropometric variables, as well as between the protein levels of ACE, ACE2, Ang 2, and Ang 1-7, and the cytokines IL-6, IL-4, IL-10, IL-13, and TNF-α which were plotted using both GraphPad Prism 8.0.2 and in a heat map in the R terminal programming package (X64 4.0.2). Furthermore, a multiple linear regression analysis was performed to investigate the associations between hepatic protein concentrations of ACE, ACE2, Ang 2, Ang 1-7, and cytokines (TNF-α, IL-6, IL-4, IL-13, and IL-10) using the R Studio programming package. Subsequent development of multiple linear regression model to determine the best predictors derived from the variables. Statistical significance was established with a *p*-value less than 0.05.

## 3. Results

Table 1 provides the means and standard deviations of anthropometric variables and biochemical values stratified by the presence or absence of SAH for the analyzed patient samples. Statistically significant differences between the groups in the anthropometrical variables such as age (*p* = 0.0146) and T2D prevalence (*p* = 0.0391), showing the group with SAH is slightly older and with a higher prevalence of T2D than the one with no SAH. We also found differences in biochemical parameters such as aPTT (*p* = 0.0197), TB (*p* = 0.0073), and IB (*p* = 0.0019); however, these values fall within the normal range and do not impact subsequent analyses, signifying homogeneity between the groups.

No statistically significant correlations were found between the biochemical and anthropometric data and hepatic protein levels of the RAS pathway components. This finding suggests that variations in biochemical and anthropometric parameters are not directly associated with the concentrations of RAS pathway proteins in the liver in our study sample.

**Figure 1:**
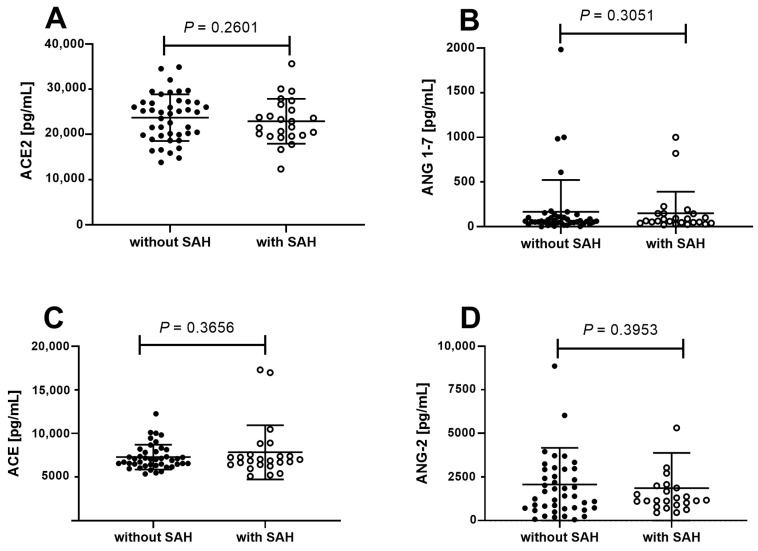
Levels of molecules from the RAS pathway in patients with morbid obesity, MASLD, and SAH compared to patients with morbid obesity and MASLD without SAH. The study population was divided based on the presence or absence of SAH. The proteins ACE (**A**) and ACE2 (**C**), along with the peptides ANG1-7 (**B**) and ANG-2 (**D**), were assessed. The analysis did not reveal any statistically significant differences between the groups.

Considering that an imbalance in the RAS characterizes SAH, we decided to investigate whether there are differences in hepatic RAS components between patients with SAH and those without SAH. Our initial hypothesis postulated that there would be significant differences in the hepatic levels of RAS molecules (ACE, ACE2, Ang 2, and Ang 1-7) between these two groups of patients. Contrary to our hypothesis, the comparative analysis did not reveal statistically significant differences in the levels of these hepatic RAS molecules between patients with morbid obesity, MASLD, and SAH and those with morbid obesity, MASLD without SAH (Figure 1A–D).

**Figure 2:**
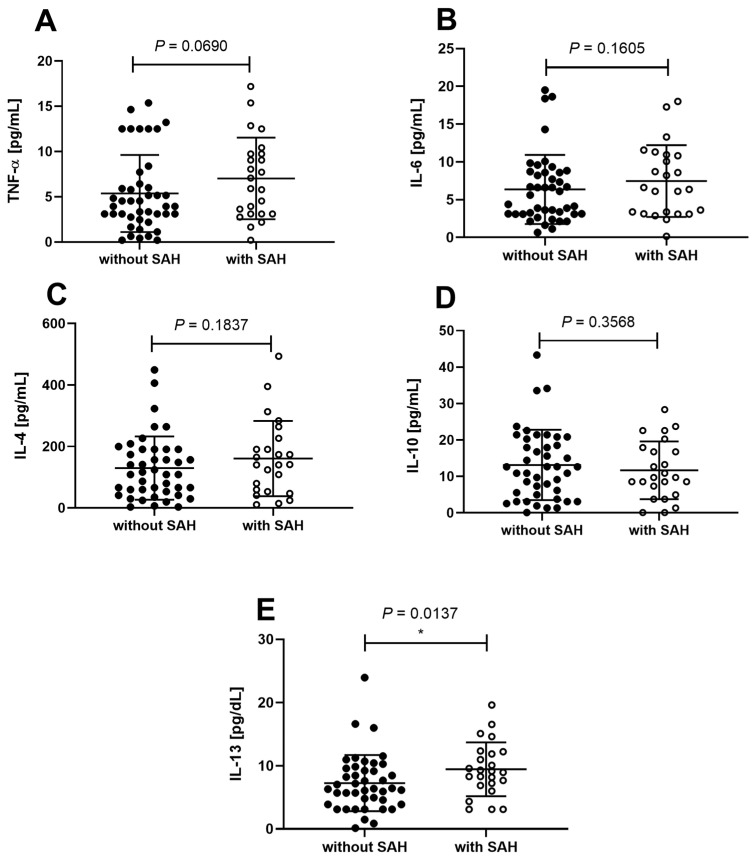
Differences in cytokine levels in patients with morbid obesity, MASLD, and SAH compared to patients without SAH. The levels of TNF-α (**A**), IL-6 (**B**), IL-10 (**C**), IL-4 (**D**), and IL-13 (**E**) in the liver were measured. Among these cytokines, only IL-13 showed a statistically significant difference between the two groups. It was found to be higher in patients with morbid obesity, MASLD, and SAH. *: *p* < 0.05.

Assuming that inflammation may be the underlying mechanism in the relationship between MASLD and hypertension, we evaluated cytokines (TNF-α, IL-6, IL-10, IL-4, and IL-13) in the liver of patients with morbid obesity, MASLD, and SAH compared to patients without SAH. We observed significant differences in the levels of the Th2 cytokine IL-13 in the liver (Figure 2E). This suggests that patients with MASLD and SAH have higher levels of this protein than those without SAH. Additionally, other cytokines showed a trend towards statistical significance, including another Th2 response cytokine, IL-4 (*p* = 0.1837) (Figure 2D), TNF-α (*p* = 0.0690) (Figure 2A), and IL-6 (*p* = 0.0599) (Figure 2B).

**Figure 3:**
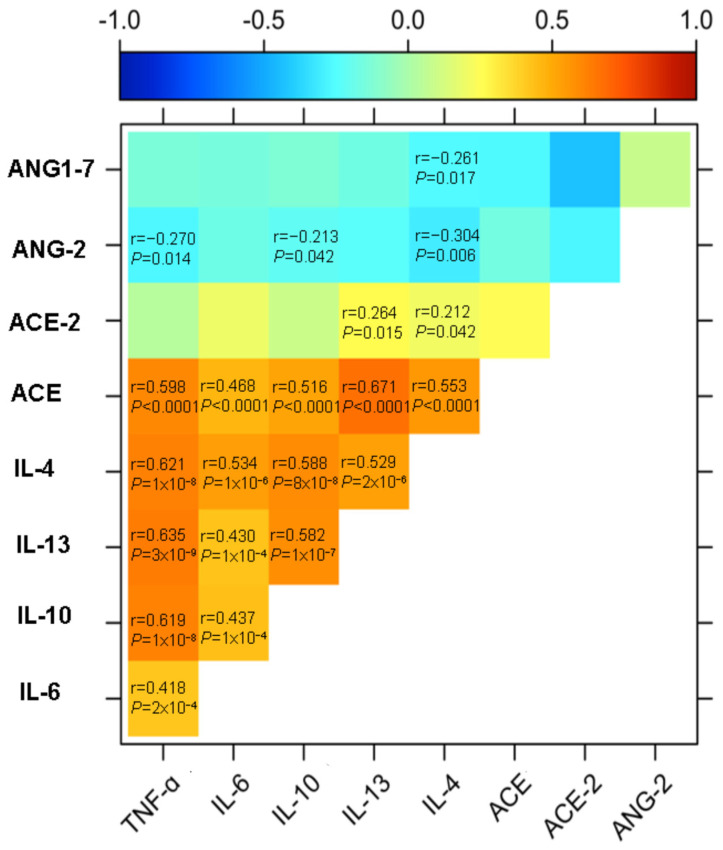
Heat map summarizing the correlations found between molecules of the RAS pathway and the cytokine profile in the liver of patients with obesity and MASLD. Inverse correlations are shown between RAS pathway peptides (Ang 2 and Ang 1–7) and cytokines, as well as strong correlations between ACE and the complete cytokine profile. ACE2 protein only correlated with Th2 response cytokines. Lastly, the relationships between cytokines were the strongest and most significant.

In addition, a correlation analysis was performed to examine the relationship between hepatic RAS pathway molecules (ACE, ACE2, Ang2, and Ang 1-7) and cytokines (TNF-α, IL-6, IL-4, IL-13, and IL-10) (Figure 3).

**Figure 4:**
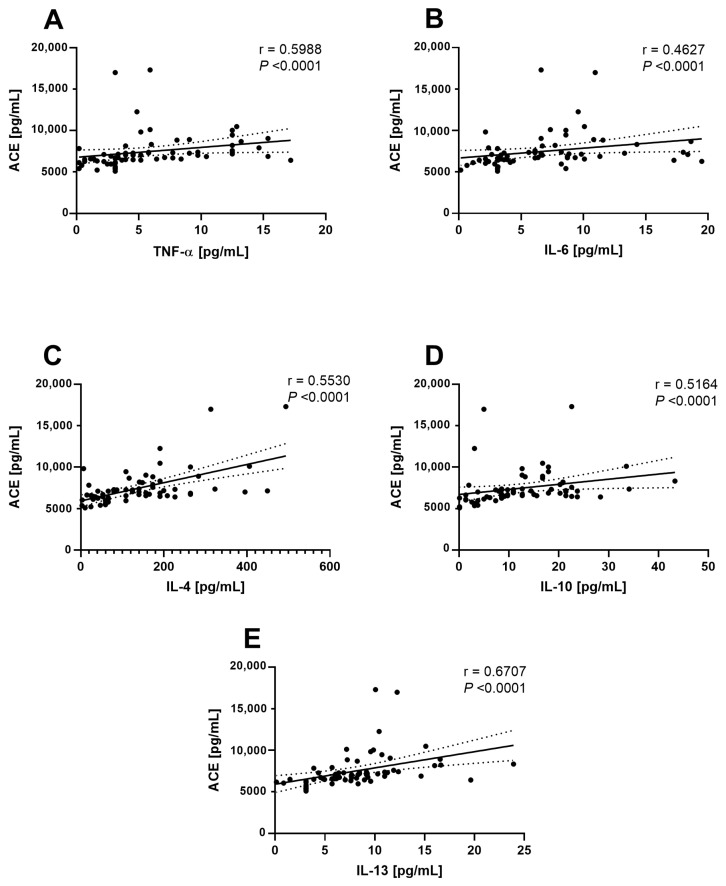
The hepatic expression of ACE strongly correlates with cytokine levels. The strong correlations between hepatic ACE and TNF-α (**A**), IL-6 (**B**), IL-4 (**C**), IL-10 (**D**), and IL-13 (**E**) are shown.

As expected, the strongest correlations were found between cytokines, as shown in Figure 3. However, a striking result is that significant solid correlations were observed between ACE and all the cytokines analyzed in this study (Figure 4); with TNF-α (r = 0.5988, *p* < 0.0001) (Figure 4A), IL-6 (r = 0.4627, *p* < 0.0001) (Figure 4B), IL-4 (r = 0.5530, *p* < 0.0001) (Figure 4C), IL-10 (r = 0.5164, *p* < 0.0001) (Figure 4D), and the strongest correlation was with IL-13 (r = 0.6707, *p* < 0.0001) (Figure 4E).

**Figure 5:**
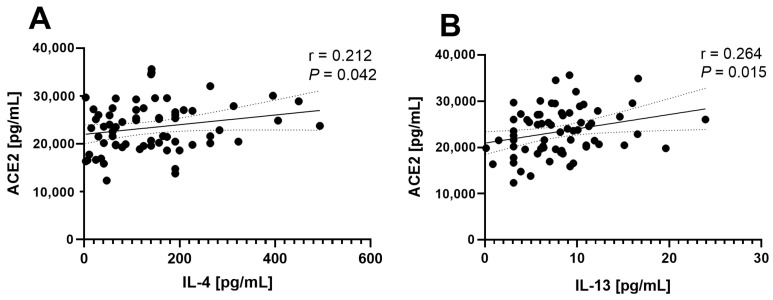
Correlation between ACE2 and cytokines in patients with obesity and MASLD. The statistically significant correlations between hepatic levels of ACE2 and IL-4 (**A**) and IL-13 (**B**) are shown.

Conversely, ACE2, a protein from the alternative RAS, showed weaker correlations, but interestingly only with IL-4 (r = 0.212, *p* = 0.042) and IL-13 (r = 0.264, *p* = 0.015) (Figure 5). These findings suggest a significant role of Th2-type cytokines in the direct or indirect regulation of ACE and ACE2 protein expression.

**Figure 6:**
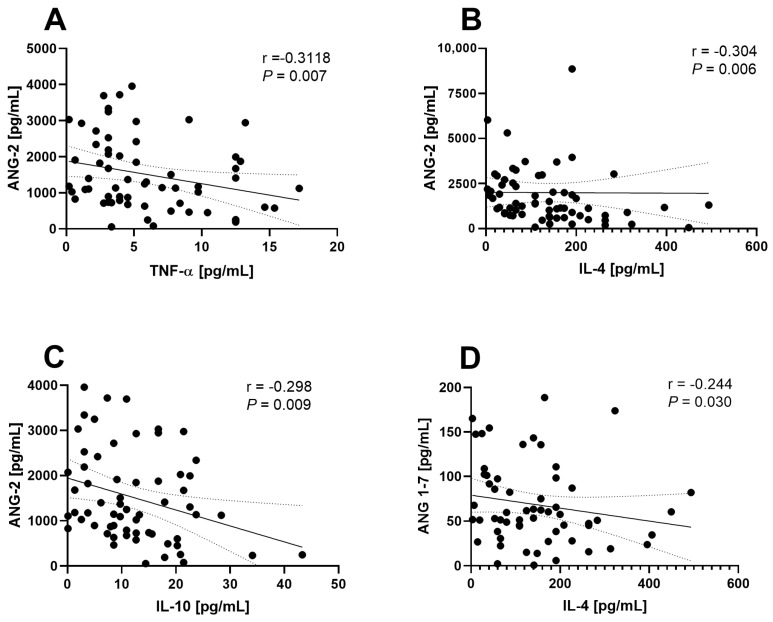
Correlation between the hepatic peptides Ang1-7 and Ang-2 with cytokines in patients with obesity and MASLD. Ang-2 showed inverse correlations with (**A**) TNF-α, (**B**) IL-4, and (**C**) IL-10, whereas Ang1–7 showed a negative correlation with (**D**) IL-4.

Moreover, an inverse correlation was identified between Ang 2 levels and the cytokines TNF-α (r = −0.3118, *p* = 0.007), IL-4 (r = −0.304, *p* = 0.006), and IL-10 (r = −0.298, *p* = 0.009), as well as between Ang 1–7 and IL-4 (r = −0.244, *p* = 0.030) (Figure 6).

Given the strong relationship between cytokine protein levels and ACE, a multiple linear regression model was conducted to identify the main predictors of hepatic ACE levels in patients with morbid obesity and MASLD. The model results were statistically significant (*p* = 0.0002068), indicating that IL-4 and IL-13 are the strongest predictors of ACE levels (*p* = 7 × 10^−5^ and *p* = 0.03, respectively) (Table 2). This finding suggests that these cytokines influence ACE levels. Specifically, the analysis revealed that for every 1 pg/mL increase in IL-13, ACE levels increase by 162.66 pg/mL. Similarly, a 1 pg/mL increase in IL-4 results in a 10.50 pg/mL increase in ACE, as indicated by the β values in Table 2. These results underscore the critical influence of Th2 cytokines, particularly IL-4 and IL-13, in regulating ACE in the liver of patients with morbid obesity and MASLD. The strong dependence of ACE on these cytokines suggests that Th2-mediated inflammatory pathways may play a significant role in modulating the RAS system in these patients.

## 4. Discussion

The relationship between obesity, metabolic syndrome, and standard cardiovascular risk factors such as MASLD and SAH is bidirectional. Studies have indicated a heightened likelihood of SAH in individuals with MASLD compared to those without this liver condition, and SAH itself has been identified as an independent predictor of MASLD. Nevertheless, the precise molecular mechanism underlying this interplay remains unresolved. One hypothesis posits that Kupffer cells provoke inflammation during MASLD, releasing pro-inflammatory cytokines into the systemic circulation. These molecular mediators can modulate various signaling cascades, including the RAS pathway. In that sense, our study aimed to examine the connection between key RAS components and cytokines at the hepatic level in morbidly obese patients. To this end, we analyzed liver biopsies from patients meeting the diagnostic criteria for MASLD due to morbid obesity. Subsequent examination did not reveal a statistically significant correlation between the hepatic protein levels of RAS components and the biochemical and anthropometric data. This outcome suggests that fluctuations in the biochemical and anthropometric parameters examined were not directly linked to the liver’s concentrations of RAS pathway proteins within our study cohort. However, it is essential to interpret this finding judiciously, considering that the biochemical values were not basal due to the necessity of stabilizing them for the patients’ surgical procedures; also, it is essential to consider that we did not have information about the treatments of these patients, among which there could be ACE inhibitors (ACEI) or angiotensin receptor blockers (ARBs), which could modify the levels of specific components of the RAS.

We observed a slight age difference between the groups, with patients who had SAH being older than those without SAH. However, this age difference may not be a confounding factor, as it has been reported that the risk of high systolic blood pressure (SBP) significantly increases from age 35 to 79, with a simultaneous early increase in the risk of high diastolic blood pressure (DBP). Therefore, patients from both groups are equally at risk of developing SAH [12].

SAH was the most common comorbidity associated with obesity in the study group, which aligns with epidemiological studies showing a prevalence of SAH of 42.5% in patients with BMI ≥ 30 kg/m^2^ [13]. Moreover, in patients with MASLD and SAH, a higher prevalence of T2D was found compared to those without SAH. This observation corresponds with substantial evidence indicating that elevated blood pressure (BP) levels are commonly present in individuals with T2D, partly attributable to the influence of underlying insulin resistance on the vasculature and renal function [14]. Conversely, accumulating evidence illustrates that disturbances in carbohydrate metabolism are more prevalent in hypertensive individuals, suggesting a bidirectional pathogenic relationship between T2D and SAH, which is also related to the development of MASLD.

Since an imbalance in the RAS characterizes SAH, exploring potential discrepancies in hepatic RAS components among patients with MASLD, both with and without SAH, was imperative. Our initial premise postulated significant differences in the hepatic levels of RAS molecules (ACE, ACE2, Ang 2, and Ang 1-7) between these two groups. To assess this premise, a comparative analysis of the levels of these molecules was executed in liver samples from patients with morbid obesity and MASLD, with and without SAH. The objective of this approach was to ascertain whether the presence of SAH in obese patients with MASLD was linked to specific alterations in the RAS pathway at the hepatic level. Contrary to our hypothesis, the comparative analysis did not unveil statistically significant differences in the hepatic levels of RAS molecules between patients with morbid obesity and MASLD, with and without SAH. This outcome indicates that, at least within the context of morbid obesity, hepatic levels of these RAS molecules do not vary significantly based on the presence or absence of SAH. These findings imply that dysfunction of the hepatic RAS may not be the primary distinguishing mechanism between patients with morbid obesity and MASLD who exhibit SAH and those who do not.

Literature suggests that hypertension and MASLD share several common pathophysiological mechanisms, encompassing inflammation, SNS activation, and insulin resistance [15]. These mechanisms may not exclusively manifest in the liver but in other organs and systems that regulate blood pressure and metabolism. Moreover, MASLD is associated with the onset of SAH and endothelial dysfunction and appears to be an independent risk factor for prehypertension and hypertension. The RAS is a complex system operating across multiple organs and systems, and substantial differences between patients with and without hypertension may be discerned in other tissues, such as the kidneys, heart, or adipose tissue, which should also be evaluated in further studies. Hence, forthcoming studies should incorporate healthy controls, lean MASLD patients, and SAH patients without other comorbidities.

Regarding the correlation analyses of RAS components with hepatic cytokines in individuals with obesity and MASLD, a significant positive correlation was observed between ACE, and all analyzed cytokines (TNF-α, IL-6, IL-4, IL-13, IL-10). This suggests that as ACE levels increase, so do the levels of these cytokines. Additionally, ACE2 showed a notable positive correlation only with IL-4 and IL-13. This implies that higher levels of ACE2 correspond to elevated levels of these Th2-type cytokines, suggesting a possible regulatory role of ACE2 in hepatic inflammation and cytokine dynamics within the RAS.

Unexpectedly, an inverse correlation was found between Ang 2 levels and TNF-α, IL-4, and IL-10. This suggests that as the levels of these cytokines increase, Ang 2 levels decrease, indicating a possible counter-regulatory mechanism between Ang 2 and these cytokines. Similarly, Ang 1-7 showed an inverse correlation with IL-4, suggesting a potential anti-inflammatory role. All these relationships underscore the complex interaction between inflammatory mediators and RAS components in hepatic inflammation. However, contrary to what has been observed in this study, Ang 2, a product of ACE enzymatic activity, has been associated with promoting Th1 and Th17 responses while suppressing Th2 responses [16]. However, the literature reports inverse correlations between Ang 2 and IL-4 [16,17], which aligns with our findings. Despite certain discrepancies with previous studies, our results are significant as they represent the first hepatic analysis in patients with obesity and MASLD regarding the relationship between cytokines and RAS components. Furthermore, this study pioneers identifying robust correlations highlighting the relationship between Th2-type responses and hepatic RAS.

The multiple linear regression model results reveal a significant relationship between hepatic levels of ACE and the cytokines IL-4 and IL-13 in patients with morbid obesity and MASLD. These findings indicate that IL-4 and IL-13 are critical predictors of ACE levels in this patient group, suggesting a crucial role for Th2-mediated inflammatory responses in modulating the RAS system in the context of obesity and MASLD.

The changes in blood flow and endothelial dysfunction in the liver microcirculation may be partially explained by increased levels of inflammatory mediators such as TNF-α, IL-1β, and chemokines [18,19]. Another contributing factor to liver endothelial dysfunction is the liver kinase B1 (LKB1), a tumor suppressor that regulates endothelial nitric oxide synthase activity, endothelial function, and blood pressure by modulating AMP kinase-mediated caveolin-1 expression [20]. Additionally, our findings suggest a potential connection between Th2 response and the modulation of liver endothelial cells, which may contribute to the development of HAS.

Th2 cytokines, including IL-4 and IL-13, play pivotal roles in the immune response. They are primarily involved in promoting anti-inflammatory and humoral immune responses, helping to counterbalance the effects of Th1 cytokines, which are typically pro-inflammatory [21]. Understanding the role of Th2 cytokines in the context of our study is critical, as it aligns with existing knowledge about the protective role of the ACE-2/Ang 1-7 axis during inflammation. This axis is known to mitigate vascular dysfunction by inhibiting VCAM-1, MCP-1, and pro-inflammatory markers such as IL-6, TNF-α, and reactive oxygen species. In the context of our study, it is suitable that Th2 responses, characterized by IL-4 and IL-13, may sustain the expression of both ACE and ACE2, thereby influencing the RAS pathway [17]. The interrelationship between these components is complex. Ang 1-7, derived from Ang 1-9 via ACE and degraded from Ang 1-9 by ACE, while ACE2 converts Ang I to Ang 1-9, can act as a modulator of Th2 cytokines [17]. This suggests that the loop involving ACE, ACE2, Ang 1-7, and Th2 responses may be a protective mechanism that helps modulate the pro-inflammatory effects of the activated RAS, thereby reducing vascular damage and hypertension.

Our findings underscore the importance of Th2-mediated responses in regulating hepatic ACE levels, highlighting a potentially novel aspect of RAS modulation in patients with obesity and MASLD. Further investigation is necessary to explore this regulatory loop further, potentially yielding new insights into therapeutic approaches targeting RAS components and inflammatory pathways in this patient demographic.

## 5. Conclusions

These findings not only contribute to understanding the underlying mechanisms of liver disease in obesity but also suggest new avenues for research and the development of therapeutic strategies targeting these specific inflammatory pathways. Despite the limitations of this study, our findings highlight the crucial role of Th2 inflammatory responses, especially IL-4 and IL-13, in regulating hepatic RAS in obese patients. These results deepen our understanding of the pathophysiological mechanisms underlying liver disease in obesity and offer new perspectives for developing therapies targeting these specific inflammatory pathways. A multidisciplinary approach that includes biochemical analyses and clinical studies would allow for more effective identification of potential biomarkers and therapeutic strategies to manage MASLD and SAH conditions.

## Figures and Tables

**Table 1:** Anthropometric and biochemical parameters of the population study.

Parameter	Whole Population *n* = 71	^a^ Without SAH *n* = 46	^b^ With SAH *n* = 25	*p* (^a^*vs.*^b^)
Age (years)	39.01 ± 10.70	39.96 ± 9.60	43.25 ± 11.69	0.0146
BMI (kg/m^2^)	43.64 ± 12.16	43.10 ± 9.54	47.11 ± 12.85	0.0680
T2D prevalence (%)	18%	15%	40%	0.0391
Serum glucose (mg/dL)	99.88 ± 20.46	99.69 ± 23.14	99.31 ± 15.95	0.2044
TC (mg/dL)	171.00 ± 41.02	172.3 ± 36.06	167.4 ± 46.43	0.3593
TGS (mg/dL)	159.11 ± 61.10	162.2 ± 58.18	152.2 ± 66.62	0.2420
HDL-C (mg/dL)	40.68 ± 15.15	40.35 ± 16.68	40.81 ± 12.84	0.3341
LDL-C (mg/dL)	120.43 ± 32.48	121.3 ± 28.35	116.4 ± 35.13	0.3217
Urea (mg/dL)	31.01 ± 12.13	29.55 ± 12.62	32.68 ± 11.21	0.0762
Creatinine (mg/dL)	0.75 ± 0.13	0.76 ± 0.14	0.73 ± 0.14	0.2237
Uric acid (mg/dL)	6.52 ± 2.01	6.37 ± 2.14	6.72 ± 1.89	0.2743
PT (s)	12.28 ± 3.04	11.67 ± 1.60	13.20 ± 4.23	0.1041
INR	0.98 ± 0.17	0.948 ± 0.08	1.041 ± 0.25	0.0946
aPTT (s)	23.30 ± 6.41	21.78 ± 6.52	23.75 ± 5.36	0.0197
Hb (g/dL)	14.85 ± 1.29	14.78 ± 1.32	15.05 ± 1.26	0.2783
Leuc (×10^3^ /µL)	8.91 ± 5.17	8.43 ± 2.091	9.65 ± 7.73	0.4295
Pla (×10^3^ /µL)	294.21 ± 71.02	304.9 ± 72.40	277.1 ± 67.27	0.1013
AP (IU/L)	81.03 ± 23.33	82.22 ± 20.47	83.41 ± 21.53	0.4475
GGT (IU/L)	37.88 ± 27.96	40.77 ± 27.01	34.77 ± 30.03	0.1262
TB (mg/dL)	0.62 ± 0.32	0.54 ± 0.27	0.74 ± 0.37	0.0073
DB (mg/dL)	0.17 ± 0.22	0.13 ± 0.06	0.24 ± 0.33	0.0777
IB (mg/dL)	0.51 ± 0.30	0.44 ± 0.24	0.64 ± 0.36	0.0012
Alb (mg/dL)	4.25 ± 0.30	4.23 ± 0.31	4.29 ± 0.29	0.2239
AST (IU/L)	26.62 ± 16.41	33.77 ± 23.58	44.87 ± 41.77	0.1839
ALT (IU/L)	42.97 ± 50.82	23.17 ± 8.27	26.95 ± 11.40	0.0868

^a^ Group of patients without SAH, and ^b^ group of patients with SAH. Abbreviations: BMI = Body Mass Index; T2D = Type Two Diabetes; SAH = Systemic Arterial Hypertension; TC = Total Cholesterol; TGS = Triglycerides; HDL-C = High Density Lipoproteins-Cholesterol; LDL-C = Low Density Lipoproteins-Cholesterol, PT = Protrombina Time; INR = International Normalized Ratio; aPTT = Activated Partial Thromboplastin Time; Hb = Hemoglobin; Leuc = Leukocytes counts; Pla = platelets counts; AP = Alkaline Phosphatase; Alb= Albumine, ALT = Alanine Aminotransferase; AST = Aspartate Aminotransferase, GGT = Gamma-Glutamyl transferase; TB = Total Bilirubin, DB = Direct Bilirubin; IB = Indirect Bilirubin.

**Table 2:** Multiple linear regression model to determine the main predictors of hepatic ACE levels in patients with morbid obesity and MASLD.

Variable	β (CI 2.5% to CI 97.5%)	*p*
TNF-α	−58.01 (−192.31–76.29)	0.39
IL-6	34.83 (−73.01–142.68)	0.52
IL-10	−46.24 (−117.24–24.75)	0.19
IL-13	162.66 (15.87–309.45)	0.03 *
IL-4	10.50 (5.59–15.42)	7.17 × 10^−5^ ***
ACE2	5.85 (−90.77–102.48)	0.90
ANG 2	0.06 (−0.17–0.29)	0.60
ANG 1-7	−0.62 (−2.13–0.88)	0.41

A multiple linear regression model used the following criteria: CI: 2.5–97.5%, residual standard error: 1810 with 58 degrees of freedom. Multiple R-squared: 0.3898, Adjusted R-squared: 0.3057, F-statistic: 4.632 with 8 and 58 degrees of freedom, *p* = 0.0002068. β, beta value; CI: confidence interval. *: *p* < 0.05, ***: *p* < 0.001.

## Data Availability

Data are available upon request.

## References

[1] Zhao Y.C., Zhao G.J., Chen Z., She Z.G., Cai J., Li H. (2020). Nonalcoholic Fatty Liver Disease: An Emerging Driver of Hypertension. Hypertension.

[2] Le M.H., Yeo Y.H., Li X., Li J., Zou B., Wu Y., Ye Q., Huang D.Q., Zhao C., Zhang J. (2022). 2019 Global NAFLD Prevalence: A Systematic Review and Meta-analysis. Clin. Gastroenterol. Hepatol..

[3] Nakagami H. (2022). Mechanisms underlying the bidirectional association between nonalcoholic fatty liver disease and hypertension. Hypertens. Res..

[4] Méndez-García L.A., Escobedo G., Minguer-Uribe A.G., Viurcos-Sanabria R., Aguayo-Guerrero J.A., Carrillo-Ruiz J.D., Solleiro-Villavicencio H. (2022). Role of the renin-angiotensin system in the development of COVID-19-associated neurological manifestations. Front. Cell. Neurosci..

[5] Cao X., Yang F., Shi T., Yuan M., Xin Z., Xie R., Li S., Li H., Yang J.-K. (2016). Angiotensin-converting enzyme 2/angiotensin-(1-7)/Mas axis activates Akt signaling to ameliorate hepatic steatosis. Sci. Rep..

[6] Silva A.C.S., Miranda A.S., Rocha N.P., Teixeira A.L. (2017). Renin angiotensin system in liver diseases: Friend or foe?. World J. Gastroenterol..

[7] Song L.N., Liu J.Y., Shi T.T., Zhang Y.C., Xin Z., Cao X., Yang J. (2020). Angiotensin-(1-7), the product of ACE2 ameliorates NAFLD by acting through its receptor Mas to regulate hepatic mitochondrial function and glycolipid metabolism. FASEB J..

[8] Yim H.E., Yoo K.H. (2008). Renin-Angiotensin System—Considerations for Hypertension and Kidney. Electrolytes Blood Press..

[9] De Albuquerque D.A., Saxena V., Adams D.E., Boivin G.P., Brunner H.I., Witte D.P., Singh R.R. (2004). An ACE inhibitor reduces Th2 cytokines and TGF-β1 and TGF-β2 isoforms in murine lupus nephritis. Kidney Int..

[10] Głuszyńska P., Lemancewicz D., Dzięcioł J.B., Hady H.R. (2021). Non-Alcoholic Fatty Liver Disease (NAFLD) and Bariatric/Metabolic Surgery as Its Treatment Option: A Review. J. Clin. Med..

[11] Rinella M.E., Lazarus J.V., Ratziu V., Francque S.M., Sanyal A.J., Kanwal F., Romero D., Abdelmalek M.F., Anstee Q.M., Arab J.P. (2023). A multisociety Delphi consensus statement on new fatty liver disease nomenclature. Hepatology.

[12] Cheng W., Du Y., Zhang Q., Wang X., He C., He J., Jing F., Ren H., Guo M., Tian J. (2022). Age-related changes in the risk of high blood pressure. Front. Cardiovasc. Med..

[13] Leggio M., Lombardi M., Caldarone E., Severi P., D’emidio S., Armeni M., Bravi V., Bendini M.G., Mazza A. (2017). The relationship between obesity and hypertension: An updated comprehensive overview on vicious twins. Hypertens. Res..

[14] Tsimihodimos V., Gonzalez-Villalpando C., Meigs J.B., Ferrannini E. (2018). Hypertension and Diabetes Mellitus Coprediction and Time Trajectories. Hypertension.

[15] Lonardo A., Nascimbeni F., Mantovani A., Targher G. (2018). Hypertension, diabetes, atherosclerosis and NASH: Cause or consequence?. J. Hepatol..

[16] Oosthuizen D., Sturrock E.D. (2022). Exploring the Impact of ACE Inhibition in Immunity and Disease. J. Renin-Angiotensin-Aldosterone Syst..

[17] Barhoumi T., Todryk S. (2023). Role of monocytes/macrophages in renin-angiotensin system-induced hypertension and end organ damage. Front. Physiol..

[18] Nasiri-Ansari N., Androutsakos T., Flessa C.M., Kyrou I., Siasos G., Randeva H.S., Kassi E., Papavassiliou A.G. (2022). Endothelial Cell Dysfunction and Nonalcoholic Fatty Liver Disease (NAFLD): A Concise Review. Cells.

[19] Castillo-Núñez Y., Almeda-Valdes P., González-Gálvez G., Arechavaleta-Granell R.M.d. (2024). Metabolic dysfunction-associated steatotic liver disease and atherosclerosis. Curr. Diab. Rep..

[20] Zhang W., Wang Q., Wu Y., Moriasi C., Liu Z., Dai X., Wang Q., Liu W., Yuan Z.-Y., Zou M.-H. (2014). Endothelial cell-specific liver kinase B1 deletion causes endothelial dysfunction and hypertension in mice in vivo. Circulation.

[21] Kokubo K., Onodera A., Kiuchi M., Tsuji K., Hirahara K., Nakayama T. (2022). Conventional and pathogenic Th2 cells in inflammation, tissue repair, and fibrosis. Front. Immunol..

